# Association of Parent-Reported Sleep Problems in Early Childhood With Psychotic and Borderline Personality Disorder Symptoms in Adolescence

**DOI:** 10.1001/jamapsychiatry.2020.1875

**Published:** 2020-07-01

**Authors:** Isabel Morales-Muñoz, Matthew R. Broome, Steven Marwaha

**Affiliations:** 1 Department of Public Health Solutions, National Institute for Health and Welfare, Helsinki, Finland; 2 Institute for Mental Health, School of Psychology, University of Birmingham, Birmingham, United Kingdom; 3 The Barberry National Centre for Mental Health, Birmingham, United Kingdom

## Abstract

**Importance:**

Persistent nightmares in childhood have been prospectively associated with psychosis and borderline personality disorder (BPD) in adolescence. However, the extent to which this association is also true for behavioral sleep problems is still unknown, and the potential mechanisms are unexplored.

**Objective:**

To examine the prospective associations between several parent-reported sleep problems in early childhood and psychotic and BPD symptoms at 11 to 13 years of age and the potential mediation of the associations by depression at 10 years of age.

**Design, Setting, and Participants:**

This cohort study assessed 13 488 participants in the Avon Longitudinal Study of Parents and Children birth cohort who were followed up for more than 13 years. Pregnant women from Avon, United Kingdom, with expected dates of delivery from April 1, 1991, to December 31, 1992, were invited to take part in the study. Data analysis was conducted from May 1 to December 31, 2019.

**Main Outcomes and Measures:**

Psychotic experiences at 12 to 13 years of age were assessed using the Psychosis-Like Symptom Interview, and BPD symptoms at 11 to 12 years of age were tested using the UK Childhood Interview for *DSM-IV* Borderline Personality Disorder. Parent-reported nighttime sleep duration, night awakening frequency, bedtime, and regularity of sleep routines were assessed when the child was 6, 18, and 30 months and 3.5, 4.8, and 5.8 years of age.

**Results:**

Data were available on 7155 participants (3718 girls [52%]) who reported on BPD symptoms and 6333 (3280 boys [52%]) who reported on BPD symptoms. Higher night awakening frequency at 18 months of age (odds ratio [OR], 1.13; 95% CI, 1.01-1.26) and less regular sleep routines at 6 months (OR, 0.68; 95% CI, 0.50-0.93), 30 months (OR, 0.64; 95% CI, 0.44-0.95), and 5.8 years (OR, 0.32; 95% CI, 0.19-0.53) of age were significantly associated with psychotic experiences in adolescence, whereas shorter nighttime sleep duration (OR, 0.78; 95% CI, 0.66-0.92) and later bedtime at 3.5 years of age (OR, 1.32; 95% CI, 1.09-1.60) were significantly associated with BPD symptoms. Results of mediation analysis were consistent with all these associations, except for later bedtime at 3.5 years and BPD in adolescence, which had no association. Depression at 10 years of age mediated the associations between frequent night awakenings at 18 months of age (bias-corrected estimate, −0.005; 95% CI, −0.008 to −0.002; *P* = .002) and irregular sleep routines at 5.8 years of age (bias-corrected estimate, −0.006; 95% CI, −0.010 to −0.003; *P* = .003) with psychosis.

**Conclusions and Relevance:**

The findings suggest that some behavioral sleep problems in childhood are distinctively associated with the onset of psychosis and BPD in adolescence, following different pathways. Furthermore, depression at 10 years of age may mediate only the association with psychosis. These findings contribute to the design of more personalized interventions in psychosis and BPD.

## Key Points

**Question:**

What is the association of early childhood sleep problems with psychosis and borderline personality disorder in adolescence?

**Findings:**

In this cohort study of data from 7155 participants, frequent night awakenings at 18 months and irregular sleep routines at 6 and 30 months and 5.8 years of age were associated with psychotic experiences, whereas shorter nighttime sleep duration and later bedtime at 3.5 years of age were associated with borderline personality disorder symptoms. Depression at 10 years of age mediated only the associations between frequent night awakenings at 18 months of age and irregular sleep routines at 5.8 years of age with psychosis.

**Meaning:**

The findings suggest that specific early childhood sleep problems are differentially associated with later psychopathologic symptoms.

## Teaser

This cohort study of UK parents and children examines the association of early childhood sleep problems with psychosis and borderline personality disorder in adolescence.

## Introduction

Theoretical and empirical research indicate adolescence as a key developmental period to study the onset of many mental disorders,^1,2^ including psychosis^3^ and borderline personality disorder (BPD),^4^ because of brain and hormonal changes occurring during this period.^5,6^ It is crucial to identify relevant factors associated with increased risk of psychopathologic symptoms among adolescents to develop effective interventions and to identify those at high risk. Sleep is a key factor associated with developmental psychopathologic symptoms^6,7,8^ and is considered a fundamental operating state of the central nervous system, occupying up to one-third of human life.^9^ Sleep may be one of the most important basic dimensions of brain function and mental health.^10,11^ Adequate sleep in childhood is essential for optimal cognitive and emotional functioning,^12,13^ and the potential association of sleep with frontal lobe functions is especially relevant in early childhood, when the brain shows substantial dynamic plasticity.^14^ Of interest, early behavioral sleep problems may be modifiable risk factors associated with future psychopathologic symptoms.^15^ This finding supports the necessity to examine the association of childhood sleep with mental disorders, such as psychosis and BPD, during adolescence.

To determine whether sleep problems precede the development of these mental disorders, prospective studies examining sleep in childhood are needed.^16^ Although there is extensive evidence that supports cross-sectional associations of sleep with BPD^17^ and psychosis,^18^ it is still unclear whether sleep problems precede their onset. Thus far, only 2 studies have longitudinally reported that children and adolescents experiencing nightmares have more psychotic experiences at 12 years^19^ and 18 years^20^ of age. With regard to BPD, only 1 study^21^ found associations between persistent nightmares across childhood and BPD symptoms at 11 to 12 years of age. One explanation might be that sleep problems indirectly increase the risk of psychosis and/or BPD by increasing the risk of depression; thus, depression could represent a mediator of these associations. Sleep disturbances are ubiquitous in depression,^22^ and childhood sleep problems are associated with subsequent depression.^23^ Other explanations might be that sleep problems are associated with both disorders, appearing earlier in development than other psychopathologic symptoms, and/or that sleep and both psychosis and BPD share common underlying mechanisms.

Although recent longitudinal research^19,20,21^ indicates that childhood nightmares are associated with the development of adolescent psychosis and BPD, those studies focused only on parasomnias, whereas the associations of more frequent sleep problems (ie, behavioral sleep problems) in early childhood, such as short sleep and frequent night awakenings, or inappropriate sleep practices have not been investigated. Given that 15% to 30% of children younger than 5 years experience behavioral sleep problems,^24^ there is a need to understand these sleep problems.^25^ In addition, psychotic symptoms are common among adults^26^ and adolescents^27^ with BPD, and genetic overlap exists.^28^ However, it is unclear whether sleep disturbances in childhood have a similar association with both conditions because of their overlap or whether sleep might have a different pathway in psychosis compared with BPD.

Identifying childhood sleep patterns and the specific time points that distinguish between psychosis and BPD may help improve our understanding of their origin. To our knowledge, no studies have examined the mediating role of depression in the association between childhood sleep and adolescent psychotic experiences and BPD symptoms. Such studies are needed to understand the potential mechanisms underlying these associations. In this study, we examined the associations between several behavioral sleep problems during specific time points in early childhood and psychotic and BPD symptoms in adolescence. We also investigated whether depression at 10 years of age mediated any associations. We hypothesized that behavioral sleep problems in early childhood would be similarly associated with psychotic experiences and BPD symptoms and that depression at 10 years of age would mediate these associations.

## Methods

### Participants

This cohort study used data from the Avon Longitudinal Study of Parents and Children (ALSPAC), a UK birth cohort study that examines the factors associated with development, health, and disease during childhood and beyond.^29,30^ Pregnant women from Avon, United Kingdom, with expected dates of delivery from April 1, 1991, to December 31, 1992, were invited to take part in the study. The ALSPAC website contains details of all the data available through a fully searchable data dictionary and variable search tool.^31^ Further details of this cohort are described in the eAppendix in the Supplement. All participants provided written informed consent, and all data were deidentified. Ethical approval was obtained from the ALSPAC law and ethics committee and the local research ethics committees. Data analysis was conducted from May 1 to December 31, 2019.

### Outcomes

The Psychosis-Like Symptom Interview, which is a semistructured face-to-face interview^32^ with 12 core questions about key psychotic experiences that have occurred since the age of 12 years, was used for the assessment of psychotic experiences at 12 to 13 years of age. We coded the presence of at least 1 definite psychotic symptom not attributable to sleep or fever.^33^

BPD symptoms at 11 to 12 years of age were assessed using a face-to-face semistructured interview: the UK Childhood Interview for *DSM-IV* Borderline Personality Disorder.^34^ The derived dichotomous outcome represented the frequent or repeated occurrence of 5 or more BPD symptoms.^35,36^

### Factors Associated With Psychotic or BPD Symptoms

Parent-reported nighttime sleep duration, night awakenings frequency, bedtime, and sleep routine regularity were assessed when children were 6, 18, and 30 months and 3.5, 4.8, and 5.8 years of age. We selected all available time points that covered infancy, toddlerhood, and preschool (ie, 6 months until 5.8 years of age). This assessment was based on our aim to identify the main time frame in early childhood that might be associated with psychotic and/or BPD symptoms in adolescence. Mothers were asked about their child’s bedtime and wake-up time and frequency of night awakenings and whether their child had regular sleep routines. Nighttime sleep duration was operationalized as the subtraction of bedtime minus wake-up time.

### Mediation Analysis

Depressive symptoms during the past 2 weeks were assessed using the short (13-item) Mood and Feelings Questionnaire.^37^ Total Mood and Feelings Questionnaire scores at 10 years of age were obtained.

### Confounders

Multiple family risk factors were assessed using the Family Adversity Index during pregnancy (long index), at 2 years of age (long index), and at 4 years of age (short index). The Family Adversity Index comprises 18 items (ie, long index) on childhood adversity and socioeconomic status. The short index excludes social, practical, and financial support. Points were summed at each time point for a total Family Adversity Index score across the 3 time points. Childhood physical and sexual abuse was reported by the mother when children were 1.5, 3.5, 4.8, 5.8, and 6.8 years of age. We coded this as yes or no at any time point. Emotional temperament was examined using the Carey Temperament Scale^38^ when children were 2 years of age. In accordance with a recent study,^21^ the mood and intensity subscales were chosen because they map most closely onto emotional temperament.^39,40^ Total scores from these 2 subscales were summed. In addition, the child’s sex, prematurity (yes vs no), and the maternal age when the infant was born were included as confounders. All these cofounders were selected based on their direct associations with our main outcomes based on previous studies.^21,41,42,43,44,45,46,47^ A description of the variables and specific time points included in this study are given in eTable 1 in the Supplement. A diagram of all the variables is given in the eFigure in the Supplement.

### Statistical Analysis

Because 57.1% of the original sample was unavailable for follow-up at 11 to 12 years of age, we conducted logistic regressions to identify significant factors associated with attrition. Adolescents lost to attrition were more often boys and had higher scores in family adversity and depression (eTable 2 in the Supplement). Using the variables associated with selective dropout as the factors, we fitted a logistic regression model (nonresponse vs response outcome) to determine weights for each individual using the inverse probability of response.^48,49^ The regression coefficients from this model were used to determine probability weights for the covariates in the main analyses.

A multistaged analysis plan was developed. We first ran logistic regression analyses in SPSS software, version 25 (SPSS Inc) to ascertain the unadjusted and adjusted associations between behavioral sleep problems in early childhood and subsequent psychotic experiences and BPD symptoms at 11 to 13 years of age. In model A, we tested unadjusted associations. In model B, we controlled for emotional temperament, family adversity, and childhood abuse. In model C, we additionally controlled for child’s sex, prematurity, and maternal age when the infant was born. Furthermore, all the time points of each sleep variable were included together. The 4 different sleep domains were evaluated separately. To avoid multicollinearity of repeatedly measured sleep variables, standardized residuals from linear regression models were used as explanatory variables.^50^ Because this study involved exploratory analysis of multiple separate hypotheses as opposed to repeated analyses of a single hypothesis, we did not adjust for multiple testing.^51^

To examine the potential mediating role of depression at 10 years of age, mediation models were tested using path analysis in SPSS-Amos (SPSS Inc), with maximum likelihood estimation to test the association of childhood sleep problems with psychotic experiences and BPD symptoms at 11 to 13 years of age with depression at 10 years of age as the mediating variable. Our analysis met the 3 assumptions of partial mediation analyses.^52^ We included as independent variables only sleep variables with significant associations in model C. In addition, we controlled for all the confounders and for the potential association between psychotic experiences and BPD symptoms in adolescence. We used bootstrapped bias-corrected 95% CIs and *P* values for assessing the significance of the standardized direct, indirect, and total associations. A 2-sided *P* < .05 was considered to be statistically significant. Missing data were dealt with using the full information maximum likelihood method.^53^

## Results

Data were available on 7155 participants (3718 girls [52%]) who reported on psychotic experiences at 12 to 13 years of age and 6333 (3280 girls [52%]) who reported on BPD symptoms at 11 to 12 years of age. Table 1 gives the frequencies and descriptive values of sociodemographic, sleep, and clinical variables.

**Table 1.** Sociodemographic, Risk, and Psychopathologic Variables in Psychotic Experiences and BPD Symptoms

Variable	Psychotic experiences at 12 y of age			BPD symptoms at 11 y of age		
	Yes (n = 376)	No (n = 6779)	*P* value	Yes (n = 472)	No (n = 5861)	*P* value
Sex, No. (%)						
Male	156 (41.7)	3281 (48.9)	.007	228 (48.4)	2825 (48.2)	.96
Female	218 (58.3)	3423 (51.1)		242 (51.6)	3031 (51.7)	
Premature, No. (%)	18 (4.8)	305 (4.5)	.43	29 (6.1)	286 (4.9)	.23
Childhood abuse, No. (%)	49 (14.4)	621 (10.6)	.05	52 (12.2)	602 (11.1)	.47
Psychotic symptoms at 12 y of age, No. (%)	376 (100.0)	0	>.99	64 (15.8)	222 (4.3)	<.001
BPD symptoms at 11 y of age, No. (%)	64 (22.2)	341 (6.5)	<.001	472 (100)	0	>.99
Regular sleep routines by age, No. (%)						
6 mo	259 (78.2)	4821 (85.8)	<.001	336 (85.1)	4440 (85.5)	.82
18 mo	274 (82.5)	4929 (87.5)	.01	341 (83.2)	4551 (87.5)	.02
30 mo	276 (85.7)	4898 (90.6)	.006	342 (88.6)	4693 (95.6)	.18
3.5 y	294 (91.0)	5050 (93.5)	.08	357 (90.4)	4673 (93.5)	.02
4.8 y	289 (93.2)	5074 (95.4)	.08	366 (95.3)	4693 (95.6)	.88
5.8 y	276 (89.6)	4957 (96.3)	<.001	346 (94.5)	4561 (96.2)	.52
Maternal age when infant was born, mean (SD), y	28.79 (4.74)	29.21 (4.50)	.10	28.78 (4.72)	29.17 (4.49)	.09
Family Adversity Index score, mean (SD)	5.13 (4.66)	3.76 (3.91)	<.001	4.94 (4.69)	3.74 (3.85)	<.001
Carey Temperament Scale score, mean (SD)	40.01 (8.66)	39.12 (8.37)	.06	40.43 (8.22)	39.09 (8.29)	.002
Mood and Feelings Questionnaire score	6.05 (4.38)	3.88 (3.34)	<.001	6.99 (4.60)	3.73 (3.21)	<.001
Night sleep at 6 mo of age, mean (SD), h	10.68 (1.41)	10.82 (1.32)	.08	10.77 (1.38)	10.81 (1.31)	.54
Night sleep by age, mean (SD), h						
18 mo	11.29 (1.19)	11.32 (1.02)	.63	11.28 (1.03)	11.32 (1.01)	.51
30 mo	11.22 (0.99)	11.21 (0.96)	.96	11.15 (1.00)	11.22 (0.96)	.19
3.5 y	11.26 (0.95)	11.26 (0.84)	.94	11.16 (0.97)	11.27 (0.84)	.02
4.8 y	11.38 (0.80)	11.39 (0.67)	.77	11.35 (0.72)	11.39 (0.67)	.21
5.8 y	11.22 (0.80)	11.28 (0.70)	.16	11.27 (0.71)	11.28 (0.70)	.86
Bedtime by age, mean (SD), h:min						
6 mo	20:00 (2:25)	19:55 (2:02)	.45	19:58 (2:21)	19:56 (1:59)	.74
18 mo	19:53 (1:04)	19:43 (1:02)	.005	19:49 (0:57)	19:44 (0:58)	.08
30 mo	19:56 (1:02)	19:45 (1:17)	.009	19:44 (1:40)	19:45 (1:15)	.74
3.5 y	19:44 (1:24)	19:42 (1:00)	.58	19:46 (1:17)	19:42 (0:59)	.23
4.8 y	19:47 (0:46)	19:42 (0:49)	.11	19:45 (0:42)	19:42 (0:44)	.22
5.8 y	19:55 (0:41)	19:49 (0:58)	.07	19:54 (0:43)	19:48 (1:01)	.12
Night awakenings by age, mean (SD)						
6 mo	0.66 (1.39)	0.59 (1.31)	.32	0.48 (1.12)	0.59 (1.25)	.09
18 mo	0.95 (1.15)	0.77 (1.04)	.002	0.83 (1.05)	0.78 (1.06)	.44
30 mo	0.70 (0.77)	0.65 (0.73)	.21	0.70 (0.76)	0.65 (0.74)	.20
3.5 y	0.73 (1.10)	0.56 (0.81)	<.001	0.63 (0.87)	0.56 (0.82)	.10
4.8 y	0.44 (0.74)	0.48 (0.33)	.83	0.42 (0.76)	0.48 (0.45)	.72
5.8 y	0.41 (1.15)	0.31 (0.43)	.50	0.38 (0.99)	0.28 (0.78)	.40

Abbreviation: BPD, borderline personality disorder.

The associations from the logistic regressions between childhood sleep and psychotic experiences appear in Table 2. In model A, later bedtime at 6 (odds ratio [OR], 1.13; 95% CI, 1.02-1.25; *P* = .02) and 30 (OR, 1.20; 95% CI, 1.02-1.43; *P* = .03) months of age, higher frequency of night awakenings at 18 months of age (OR, 1.14; 95% CI, 1.02-1.27; *P* = .02), and less regular sleep routines at 6 months of age (OR, 0.65; 95% CI, 0.48-0.88; *P* = .005) and 30 months of age (OR, 0.61; 95% CI, 0.42-0.88; *P* = .009) and 5.8 (OR, 0.31; 95% CI, 0.19-0.50; *P* < .001) years of age were significantly associated with psychotic experiences at 12 to 13 years of age. In model B, all these associations except for later bedtime at 6 and 30 months remained significant (night awakenings frequency at 18 months and psychotic experiences at 12-13 years: OR, 1.12; 95% CI, 1.00-1.25; *P* = .04; regular sleep routines at 6 months and psychotic experiences at 12-13 years: OR, 0.70; 95% CI, 0.51-0.95; *P* = .02; regular sleep routines at 30 months and psychotic experiences at 12-13 years: OR, 0.61; 95% CI, 0.42-0.90; *P* = .01; and regular sleep routines at 5.8 years and psychotic experiences at 12-13 years: OR, 0.34; 95% CI, 0.20-0.55; *P* < .001). The significant associations obtained in model C were the same as those in model B; the strengths of these associations were greater except for regular sleep routines at 30 months (night awakenings frequency at 18 months and psychotic experiences at 12-13 years: OR, 1.13; 95% CI, 1.01-1.26; *P* = .03; regular sleep routines at 6 months and psychotic experiences at 12-13 years: OR, 0.68; 95% CI, 0.50-0.93; *P* = .02; regular sleep routines at 30 months and psychotic experiences at 12-13 years: OR, 0.64; 95% CI, 0.44-0.95; *P* = .02; and regular sleep routines at 5.8 years and psychotic experiences at 12-13 years: OR, 0.32; 95% CI, 0.19-0.53; *P* < .001).

**Table 2.** Unadjusted and Adjusted Associations Between Childhood Sleep Patterns and Psychotic Experiences at 12 to 13 Years of Age^a^

Sleep variable	Psychotic symptoms								
	Model A			Model B			Model C		
	β	*P* value	OR (95% CI)	β	*P* value	OR (95% CI)	β	*P* value	OR (95% CI)
**Night sleep duration by age**									
6 mo	−0.045	.35	0.96 (0.87-1.05)	−0.032	.51	0.97 (0.88-1.06)	−0.043	.38	0.96 (0.87-1.06)
18 mo	0.011	.88	1.01 (0.88-1.16)	0.009	.90	1.01 (0.88-1.16)	0.006	.93	1.01 (0.88-1.16)
30 mo	−0.065	.42	0.94 (0.80-1.10)	−0.060	.45	0.94 (0.81-1.10)	−0.064	.42	0.94 (0.80-1.10)
3.5 y	0.009	.92	1.01 (0.83-1.22)	0.038	.69	1.04 (0.86-1.26)	0.024	.80	1.02 (0.85-1.24)
4.8 y	0.019	.87	1.02 (0.82-1.27)	0.010	.93	1.01 (0.81-1.26)	−0.008	.94	0.99 (0.80-1.24)
5.8 y	0.031	.79	1.03 (0.82-1.29)	0.062	.59	1.06 (0.85-1.34)	0.045	.70	1.05 (0.83-1.31)
**Bedtime by age**									
6 mo	0.119	.02	1.13 (1.02-1.25)	0.095	.07	1.10 (0.99-1.22)	0.099	.06	1.10 (1.00-1.23)
18 mo	0.108	.16	1.11 (0.96-1.30)	0.085	.28	1.09 (0.93-1.27)	0.074	.34	1.08 (0.92-1.26)
30 mo	0.187	.03	1.20 (1.02-1.43)	0.127	.13	1.10 (0.96-1.30)	0.130	.13	1.11 (1.00-1.24)
3.5 y	−0.037	.73	0.96 (0.78-1.19)	−0.068	.52	0.94 (0.76-1.15)	−0.069	.52	0.93 (0.76-1.15)
4.8 y	−0.007	.95	0.99 (0.80-1.24)	0.012	.92	1.01 (0.81-1.26)	0.020	.86	1.02 (0.82-1.28)
5.8 y	−0.116	.36	0.89 (0.70-1.14)	−0.143	.25	0.87 (0.68-1.11)	−0.112	.37	0.89 (0.70-1.14)
**Night awakenings frequency by age**									
6 mo	0.046	.32	1.05 (0.96-1.15)	0.036	.44	1.04 (0.95-1.14)	0.045	.34	1.05 (0.95-1.15)
18 mo	0.131	.02	1.14 (1.02-1.27)	0.114	.04	1.12 (1.00-1.25)	0.120	.03	1.13 (1.01-1.26)
30 mo	−0.089	.360	0.92 (0.76-1.11)	−0.082	.41	0.92 (0.76-1.12)	−0.088	.38	0.92 (0.75-1.11)
3.5 y	0.093	.29	1.10 (0.92-1.30)	0.072	.42	1.08 (0.90-1.28)	0.075	.40	1.08 (0.91-1.28)
4.8 y	−0.016	.67	0.98 (0.92-1.06)	−0.016	.69	0.98 (0.91-1.07)	−0.023	.66	0.98 (0.88-1.08)
5.8 y	0.009	.69	1.01 (0.97-1.05)	0.010	.64	1.01 (0.97-1.06)	0.010	.63	1.01 (0.97-1.05)
**Regular sleep routines by age**									
6 mo	−0.432	.005	0.65 (0.48-0.88)	−0.362	.02	0.70 (0.51-0.95)	−0.387	.02	0.68 (0.50-0.93)
18 mo	−0.276	.11	0.76 (0.54-1.07)	−0.234	.18	0.79 (0.56-1.12)	−0.247	.16	0.78 (0.55-1.11)
30 mo	−0.499	.009	0.61 (0.42-0.88)	−0.488	.01	0.61 (0.42-0.90)	−0.439	.02	0.64 (0.44-0.95)
3.5 y	−0.093	.71	0.91 (0.56-1.49)	−0.003	.99	1.00 (0.60-1.64)	0.039	.88	1.04 (0.62-1.73)
4.8 y	−0.239	.40	0.79 (0.45-1.38)	−0.203	.48	0.82 (0.46-1.44)	−0.134	.65	0.88 (0.49-1.56)
5.8 y	−1.176	<.001	0.31 (0.19-0.50)	−1089	<.001	0.34 (0.20-0.55)	−1.140	<.001	0.32 (0.19-0.53)

Abbreviation: OR, odds ratio.

^a^ All the time points are included within the same model for each sleep variable. Standardized residuals are used as sleep measures at 18 and 30 months and at 3.5, 4.8, and 5.8 years, in which the sleep variables at later measurement time points are regressed on the corresponding variables at previous measurement waves. Model A is the unadjusted model; model B, adjusted for emotional temperament at 2 years, family adversity, and childhood abuse; and model C, adjusted for emotional temperament at 2 years, family adversity, childhood abuse, sex, prematurity, and maternal age when infant was born.

The results of the logistic regressions for BPD symptoms are reported in Table 3. In model A, shorter nighttime sleep duration at 3.5 years of age (OR, 0.79; 95% CI, 0.67-0.93; *P* = .005) and later bedtime at 6 months of age (OR, 1.10; 95% CI, 1.00-1.20; *P* = .046) and 3.5 years of age (OR, 1.31; 95% CI, 1.08-1.58; *P* = .005) were significantly associated with BPD symptoms in adolescence. All results except for bedtime at 6 months remained in model B (night sleep duration at 3.5 years and BPD symptoms at 11-12 years: OR, 0.82; 95% CI, 0.70-0.97; *P* = .02; and bedtime at 3.5 years and BPD symptoms at 11-12 years: OR, 1.25; 95% CI, 1.04-1.52; *P* = .02) and model C (night sleep duration at 3.5 years and BPD symptoms at 11-12 years: OR, 0.78; 95% CI, 0.66-0.92; *P* = .004; and bedtime at 3.5 years and BPD symptoms at 11-12 years: OR, 1.32; 95% CI, 1.09-1.60; *P* = .005), and the strength of these associations was increased in model C.

**Table 3.** Unadjusted and Adjusted Associations Between Childhood Sleep Patterns and Borderline Personality Disorder Symptoms at 11 to 12 Years of Age^a^

Sleep variable	Borderline personality disorder symptoms								
	Model A			Model B			Model C		
	β	*P* value	OR (95% CI)	β	*P* value	OR (95% CI)	β	*P* value	OR (95% CI)
**Night sleep duration by age**									
6 mo	−0.035	.41	0.96 (0.89-1.05)	−0.043	.31	0.96 (0.88-1.04)	−0.040	.36	0.96 (0.88-1.05)
18 mo	−0.049	.44	0.95 (0.84-1.08)	−0.050	.42	0.95 (0.84-1.08)	−0.063	.33	0.94 (0.83-1.06)
30 mo	−0.027	.71	0.97 (0.84-1.12)	−0.011	.88	0.99 (0.86-1.14)	−0.009	.91	0.99 (0.86-1.14)
3.5 y	−0.234	.005	0.79 (0.67-0.93)	−0.193	.02	0.82 (0.70-0.97)	−0.245	.004	0.78 (0.66-0.92)
4.8 y	0.115	.25	1.12 (0.92-1.36)	0.119	.23	1.13 (0.93-1.37)	0.163	.11	1.18 (0.96-1.44)
5.8 y	0.104	.32	1.11 (0.90-1.36)	0.073	.48	1.08 (0.88-1.32)	0.051	.62	1.05 (0.86-1.29)
**Bedtime by age**									
6 mo	0.093	.046	1.10 (1.00-1.20)	0.089	.06	1.09 (1.00-1.20)	0.082	.09	1.09 (0.99-1.19)
18 mo	0.043	.55	104 (0.91-1.20)	0.050	.48	1.05 (0.92-1.21)	0.056	.43	1.06 (0.92-1.22)
30 mo	−0.079	.35	0.92 (0.78-1.09)	−0.098	.25	0.91 (0.77-1.07)	−0.088	.31	0.92 (0.77-1.08)
3.5 y	0.269	.005	1.31 (1.08-1.58)	0.227	.02	1.25 (1.48-1.52)	0.274	.005	1.32 (1.08-1.60)
4.8 y	−0.151	.15	0.86 (0.70-1.06)	−0.153	.14	0.86 (0.70-1.05)	−0.210	.053	0.81 (0.66-1.00)
5.8 y	−0.044	.69	0.96 (0.77-1.19)	−0.040	.71	0.96 (0.78-1.19)	−0.032	.77	0.97 (0.78-1.20)
**Night awakenings frequency by age**									
6 mo	−0.082	.09	0.92 (0.84-1.01)	−0.085	.08	0.92 (0.84-1.01)	−0.078	.12	0.92 (0.84-1.02)
18 mo	0.021	.69	1.02 (0.92-1.14)	−0.002	.97	1.00 (0.90-1.11)	−0.024	.68	0.98 (0.87-1.09)
30 mo	0.090	.28	1.09 (0.93-1.29)	0.088	.30	1.09 (0.93-1.29)	0.104	.22	1.11 (0.94-1.31)
3.5 y	0.096	.20	1.10 (0.95-1.28)	0.073	.34	1.08 (0.93-1.25)	0.060	.45	1.06 (0.91-1.24)
4.8 y	−0.025	.57	0.98 (0.90-1.06)	−0.033	.59	0.97 (0.86-1.09)	−0.033	.60	0.97 (0.86-1.10)
5.8 y	0.008	.64	1.01 (0.97-1.04)	0.010	.60	1.01 (0.97-1.05)	0.011	.57	1.01 (0.98-1.05)
**Regular sleep routines by age**									
6 mo	0.091	.57	1.10 (0.80-1.49)	0.140	.38	1.15 (0.84-1.58)	0.139	.40	1.15 (0.83-1.58)
18 mo	−0.223	.16	0.80 (0.58-1.10)	−0.167	.30	0.85 (0.62-1.16)	−0.154	.35	0.86 (0.62-1.19)
30 mo	0.128	.54	1.14 (0.76-1.71)	0.184	.38	1.20 (0.80-1.82)	0.179	.40	1.20 (0.79-1.82)
3.5 y	−0.428	.06	0.65 (0.41-1.02)	−0.357	.12	0.70 (0.44-1.10)	−0.343	.15	0.71 (0.44-1.13)
4.8 y	0.106	.71	1.11 (0.63-1.95)	0.156	.59	1.17 (0.67-2.05)	0.217	.46	1.24 (0.70-2.22)
5.8 y	−0.511	.06	0.60 (0.35-1.03)	−0.327	.26	0.72 (0.41-1.27)	−0.395	.17	0.67 (0.38-1.10)

Abbreviation: OR, odds ratio.

^a^ All the time points are included within the same model for each sleep variable. Standardized residuals are used as sleep measures at 18 and 30 months and at 3.5, 4.8, and 5.8 years, in which the sleep variables at later measurement time points are regressed on the corresponding variables at previous measurement waves. Model A is the unadjusted model; model B, adjusted for emotional temperament at 2 years, family adversity, and childhood abuse; and model C, adjusted for emotional temperament at 2 years, family adversity, childhood abuse, sex, prematurity, and maternal age when infant was born.

In examining whether depression at 10 years of age was a mediating factor of these associations, path analysis model fit indexes indicated excellent model fit (χ^2^ = 3.05, *P* = .80; root mean square error of approximation = 0; comparative fit index = 1.00). Consistent with the adjusted logistic regression analysis, frequent night awakenings at 18 months of age (β = 0.007, SE = 0.001, *P* < .001) and irregular sleep routines at 6 months of age (β = −0.027, SE = 0.004, *P* < .001) and 3.5 (β = −0.027, SE = 0.006, *P* < .001) and 5.8 (β = −0.83, SE = 0.008, *P* < .001) years of age were significantly associated with psychotic symptoms at 12 to 13 years of age. Short nighttime sleep at 3.5 years was associated with BPD symptoms at 11 to 12 years of age (β = −0.008, SE = 0.002, *P* < .001). However, there was not a significant association between bedtime at 3.5 years and BPD at 11 to 12 years of age. All these results remained after controlling for the association between psychosis and BPD. Direct associations are shown in the Figure, and estimates of the direct, indirect, and total effects are shown in eTable 3 in the Supplement. The significant direct associations between the covariates and the independent, mediator, and dependent variables were sex and depression (β = −0.02, *P* < .001), sex and psychosis (β = 0.19, *P* < .001), prematurity and depression (β = 0.32, *P* < .001), prematurity and BPD symptoms (β = 0.13, *P* < .001), maternal age when infant born (β = −0.03, *P* < .001), childhood abuse and depression (β = 0.43, *P* < .001), childhood abuse and BPD symptoms (β = 0.01, *P* = .04), family adversity and depression (β = 0.12, *P* < .001), family adversity and psychosis (β = 0.03, *P* < .001), family adversity and BPD (β = 0.003, *P* < .001), emotional temperament and depression (β = 0.02, *P* < .001), and emotional temperament and BPD (β = 0.001, *P* = .004). The significant associations between the covariates were maternal age when infant was born with emotional temperament at 2 years of age (β = −0.36, *P* < .001), prematurity (β = −0.03, *P* < .001), and family adversity (β = −0.11, *P* < .001); emotional temperament at 2 years of age with sex (β = −0.07, *P* = .011), prematurity (β = 0.05, *P* < .001), childhood abuse (β = 0.07, *P* < .001), and family adversity (β = 0.28, *P* < .001); sex with prematurity (β = −0.004, *P* < .001) and childhood abuse (β = −0.006, *P* < .001); and childhood abuse with family adversity (β = 0.13, *P* < .001).

**Figure.**
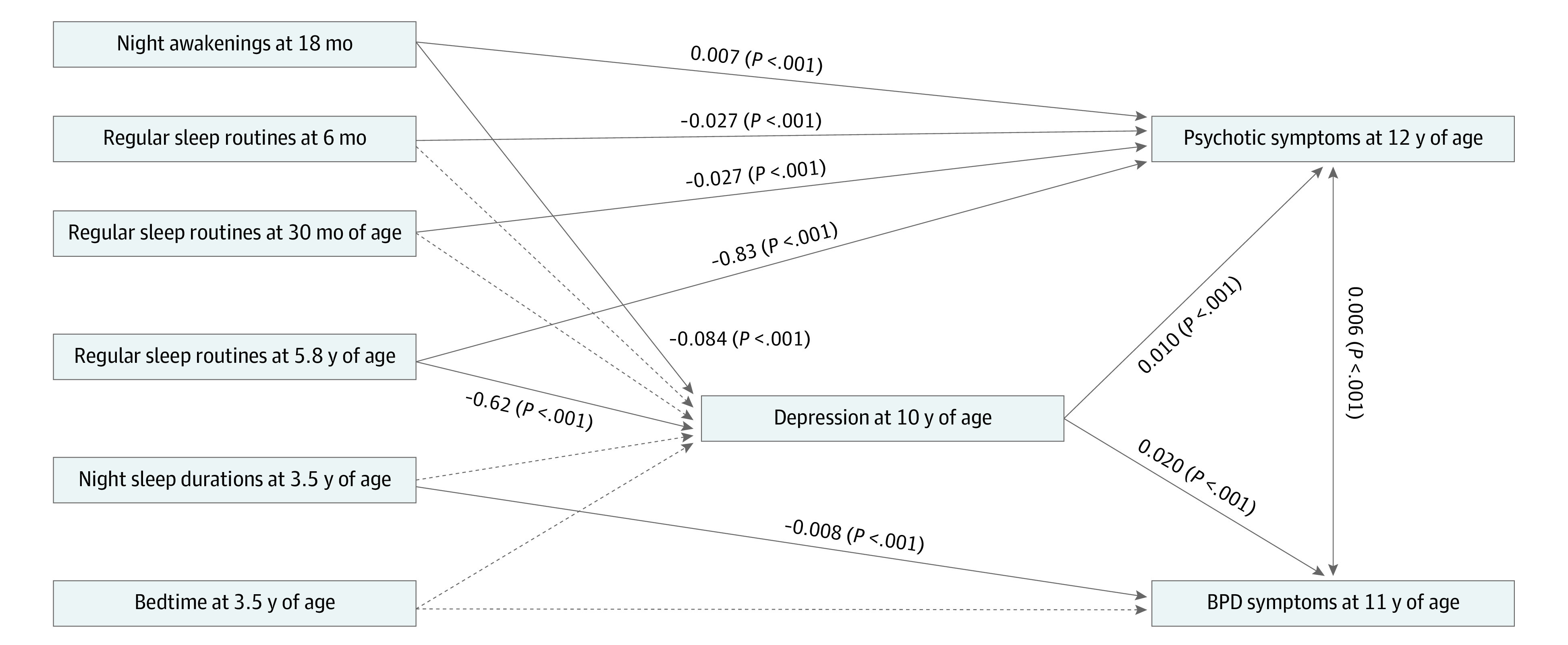
Path Diagram Showing the Main Significant Direct Associations in the Final Model This figure shows only the direct associations of the independent, mediator, and dependent variables. Night awakening at 18 months of age, regular sleep routines at 6 months and 3.5 and 5.8 years of age, night sleep duration at 3.5 years of age, and bedtime at 3.5 years of age represent the independent variables (exposures); depression at 10 years of age represents the mediator; and psychotic symptoms at 12 to 13 years of age and borderline personality disorder (BPD) symptoms at 11 to 12 years of age represent the dependent variables (outcomes). The covariates also included in this path analyses were sex, prematurity, maternal age when infant was born, childhood abuse, family adversity, and emotional temperament. Significant pathways are signified by solid arrows and nonsignificant modeled pathways by gray dotted lines.

The associations between all the covariates and BPD and psychotic symptoms were partly mediated by depression (the eAppendix in the Supplement gives estimates of total, direct, and indirect effects). However, depression only partly (ie, meeting 3 of the 4 assumptions of mediation)^52^ mediated the associations of night awakenings at 18 months of age (bias-corrected estimate, −0.005; 95% CI, −0.008 to −0.002; *P* = .002) and regular sleep routines at 5.8 years of age (bias-corrected estimate, −0.006; 95% CI, −0.010 to −0.003; *P* = .003) with later psychosis (Table 4).

**Table 4.** Bootstrapped Bias-Corrected Estimate (95% CIs) and *P* Values for the Hypothesized Indirect Pathways to Psychotic Experiences and BPD Symptoms With Depression as the Mediator

Variable	Depression at 10 y of age			
	Psychotic experiences at 12-13 y of age		BPD symptoms at 11-12 y of age	
	Estimate (95% CI)	*P* value	Estimate (95% CI)	*P* value
Family adversity	0.026 (0.021 to 0.032)	.003	0.048 (0.042 to 0.056)	.003
Childhood abuse	0.007 (0.004 to 0.011)	.003	0.013 (0.008 to 0.019)	.003
Prematurity	0.005 (0.002 to 0.008)	.002	0.009 (0.004 to 0.014)	.003
Sex	−0.006 (−0.009 to −0.004)	.006	−0.012 (−0.017 to −0.007)	.005
Maternal age when born	−0.007 (−0.011 to −0.005)	.002	−0.014 (−0.020 to −0.009)	.002
Emotional temperament 2 y of age	0.008 (0.005 to 0.011)	.005	0.014 (0.009 to 0.020)	.005
Night awakening 18 mo of age	−0.005 (−0.008 to −0.002)	.002	NA	NA
Regular sleep routines by age				
6 mo	−0.001 (−0.004 to 0.001)	.30	NA	NA
3.5 y	−0.001 (−0.002 to −0.009)	.71	NA	NA
5.8 y	−0.006 (−0.010 to −0.003)	.003	NA	NA
Night sleep duration at 3.5 y of age	NA	NA	−0.004 (−0.012 to 0.002)	.22
Bedtime at 3.5 y of age	NA	NA	0.006 (−0.001 to 0.121)	.12

Abbreviations: BPD, borderline personality disorder; NA, not applicable.

## Discussion

This is the first study, to our knowledge, to examine the prospective associations between early childhood sleep problems and adolescent psychotic experiences and BPD symptoms. We also tested whether depression at 10 years of age represents a possible mechanism by which early sleep problems are associated with psychotic experiences or BPD symptoms. Our main findings indicated that frequent night awakenings at 18 months of age and irregular sleep routines at 6 and 30 months and 5.8 years of age were associated with psychotic experiences at 12 to 13 years of age, whereas shorter nighttime sleep duration and later bedtime at 3.5 years of age were associated with BPD symptoms at 11 to 12 years of age. Depression at 10 years of age mediated only the associations between frequent night awakenings at 18 months of age and irregular sleep routines at 5.8 years of age with later psychotic experiences. In addition, contrary to an existing cross-sectional study in chronotype,^54^ we did not find significant longitudinal associations between bedtime and psychotic symptoms.

Frequent night awakenings at 18 months of age and irregular sleep routines at 6 months and 30 months and 5.8 years of age were prospectively associated with psychotic experiences at 12 to 13 years of age, even when depression at 10 years of age was included as a mediator and after controlling for BPD symptoms at 11 to 12 years of age. This finding suggests a specific pathway between these childhood sleep problems and adolescent psychotic experiences. This finding is distinct from but adds to the existing 2 studies^19,20^ that reported an association between persistent parasomnias and subsequent psychosis. In the current study, which examined how much behavioral sleep problems are specifically associated with psychotic experiences, we found that night awakenings at 18 months of age were associated with psychotic experiences in adolescence. Insomnia is common in psychosis,^55,56^ and frequent night awakening is 1 of the diagnostic criteria for insomnia.^57^ Our findings support the idea that insomnia contributes to psychosis but suggest that difficulties can appear years before the onset of psychotic experiences. We also found that irregular sleep routines across several stages of childhood were associated with psychotic experiences in adolescence. A lack of routine is a key feature of sleep problems in young people at ultrahigh risk of psychosis.^58^

Shorter nighttime sleep duration at 3.5 years of age was the only sleep variable directly associated with BPD in adolescence. Given the persistent associations after controlling for interactions with psychotic symptoms, our results suggest a separate and specific pathway for BPD. A previous study^21^ found that persistent nightmares in childhood were independently associated with BPD symptoms at 11 to 12 years of age. This was the first study, to our knowledge, to report an independent association between short sleep in preschool-aged children and BPD symptoms in adolescents, consistent with cross-sectional studies^17,59^ in which patients with BPD reported short sleep.

Depression at 10 years of age mediated the associations between frequent night awakenings at 18 months of age and irregular sleep routines at 5.8 years of age and psychotic symptoms in adolescence. Although depression is considered a mediator in the sleep-psychosis association,^56,60^ the extent to which this is true for all the sleep patterns was previously unknown, to our knowledge. Our results are supported by existing models of the genesis and maintenance of paranoia and hallucinations that consider depression to be central.^61,62,63^ Furthermore, the role of some specific neurotransmitters in the brain, such as dopamine and serotonin, might also partially explain the mediating role of depression, taking into account that both neurotransmitters are involved in not only the sleep-wake cycle^64^ but also the development of depression^65^ and psychosis.^66^

Depression at 10 years of age did not mediate the associations between short nighttime sleep at 3.5 years of age and BPD, although BPD shares common sleep and biological features with depression.^67,68^ This finding again supports the specificity of pathways. Our findings indicate that there might be a direct association between childhood sleep duration and later BPD symptoms independently of depression. Patients with BPD often stay up late and sleep during the day,^69^ indicating that altered circadian rhythms may be associated with BPD.^70^ Of interest, infants with slow circadian rhythm development sleep less during the night,^71^ suggesting that infants’ short nighttime sleep might be an indicator of circadian dysfunction. In this study, we found that shorter nighttime sleep in toddlers was prospectively associated with BPD symptoms, indicating that altered circadian rhythm might be associated with subsequent BPD symptoms. These results, however, should be interpreted with caution because circadian disturbances are commonly observed in patients with depression^72^; thus, depression might still play an important role. Another explanation might be that other factors mediate this association, such as emotion dysregulation.^40,73,74^

The current robust analyses indicate some specificity between particular early sleep difficulty and later differing psychopathologic symptoms. This finding may be a function of assessing sleep problems earlier than existing research. This finding could have important implications for helping practitioners identify children who might be at higher risk for psychotic experiences or BPD symptoms in adolescence and potentially lead to the design of more effectively targeted sleep or psychological interventions to prevent the onset of or attenuate these mental disorders.

### Strengths and Limitations

This study has several strengths. First, we used data from a large prospective cohort. Second, psychotic experiences and BPD symptoms were assessed using validated interviews. Third, we addressed sleep early in childhood.

There are also some limitations. First, the sleep variables are based on parent reports, and objective measures such as actigraphy were not available; this study focused on parent-reported perceptions of sleep, which could be different from objective sleep. However, parental sleep reports are considered valid in young children,^75^ and a previous study^76^ reported low specificity for actigraphs, particularly for infants. Second, other potential contributing factors, such as pervasive developmental delay, hyperactivity, prenatal medications, or caregiver shift-work, should be explored in future studies. In addition, we were unable to account for changes in confounding variables over time, such as within family adversity.^77^ Third, although the study design cannot determine causality, analyses meet some Bradford Hill criteria.^78^ Fourth, the ALSPAC cohort is representative of the UK population,^29,30^ which is racially and ethnically diverse. However, this diversity is not identical in characteristics or perhaps extent to that in other populations. Fifth, the degree of prematurity could be an important confounder. We first included the variable prematurity as a confounder and then replaced this with gestational age in weeks in the analyses, but this did not change the results. Sixth, we did not check for interactional effects; therefore, our path model does not account for these.

## Conclusions

In this study, frequent night awakenings at 18 months of age and irregular sleep routines at 6 months, 30 months, and 5.8 years of age were associated with psychotic experiences at 12 to 13 years of age, whereas only short nighttime sleep at 3.5 years of age was associated with BPD symptoms at 11 to 12 years of age. Furthermore, depression at 10 years of age mediated the association between night awakenings at 18 months of age and irregular sleep routines at 5.8 years of age with psychotic experiences. These findings suggest that the associations between childhood sleep and psychotic experiences as well as childhood sleep and BPD symptoms in adolescence follow different pathways. These results could contribute to the design of more personalized sleep and psychological interventions in psychosis and BPD.

## Notes

Supplement. eAppendix. Further Details of ALSPAC Cohort eTable 1. Description of the Variables, Measures, and Questionnaires Used at Each Time Point eTable 2. Differences in Sociodemographic and Clinical Variables Between Nonparticipating and Participating Subjects in the Study eTable 3. Estimates of Total, Direct, and Indirect Effects eFigure. Causal Diagram to Represent Temporal Ordering, Including Confounders
