# Supplementary material for: Association of Parent-Reported Sleep Problems in Early Childhood With Psychotic and Borderline Personality Disorder Symptoms in Adolescence
Source: JAMA Psychiatry. 2020 Jul 1;77(12):1–11. doi: 10.1001/jamapsychiatry.2020.1875 (PMC7330826; doi:10.1001/jamapsychiatry.2020.1875)
Supplement: Supplement. — eAppendix. Further Details of ALSPAC Cohort eTable 1. Description of the Variables, Measures, and Questionnaires Used at Each Time Point eTable 2. Differences in Sociodemographic and Clinical Variables Between Nonparticipating and Participating Subjects in the Study eTable 3. Estimates of Total, Direct, and Indirect Effects eFigure. Causal Diagram to Represent Temporal Ordering, Including Confounders [file jamapsychiatry-e201875-s001.pdf]

Supplementary Online Content

Morales-Muñoz I, Broome MR, Marwaha S. Association of parent-reported sleep problems in early childhood with psychotic and borderline personality disorder symptoms in adolescence. *JAMA Psychiatry.* Published online July 1, 2020. doi:10.1001/jamapsychiatry.2020.1875

eAppendix. Further Details of ALSPAC Cohort

eTable 1. Description of the Variables, Measures, and Questionnaires Used at Each Time Point

eTable 2. Differences in Sociodemographic and Clinical Variables Between Nonparticipating and Participating Subjects in the Study

eTable 3. Estimates of Total, Direct, and Indirect Effects

eFigure. Causal Diagram to Represent Temporal Ordering, Including Confounders

This supplementary material has been provided by the authors to give readers additional information about their work.

© 2020 Morales-Muñoz I et al. *JAMA Psychiatry.*

Appendix. Further Details of ALSPAC Cohort

The initial number of pregnancies enrolled was 14,541 (for these at least one questionnaire was returned or a "Children in Focus" clinic had been attended by 19/07/99). Of these initial pregnancies, there was a total of 14,676 foetuses, resulting in 14,062 live births and 13,988 children who were alive at 1 year of age. When the oldest children were approximately 7 years of age, an attempt was made to bolster the initial sample with eligible cases who had failed to join the study originally. As a result, in our study, as some variables were collected from the age of seven onwards there were data available for more than the 14,541 pregnancies mentioned above.

© 2020 Morales-Muñoz I et al. *JAMA Psychiatry.*

eTable 1. Description of the Variables, Measures and Questionnaires Used at Each Time Point

			Pregnancy	Birth	6mo	18mo	24mo	30mo	3.5y	4y	4.8y	5.8y	6.8y	10y	11-12y	12-13y
**Variable**	**Measure**	**Questionnaire**														
Sex	Parent-reported			x												
Prematurity	Parent-reported			x												
Maternal age born	Parent-reported			x												
Family adversity	Parent-reported	FAI	x				x			x						
Emotional temperament	Parent-reported	CTS					x									
Childhood abuse	Parent-reported					x			x		x	x	x			
Regular sleep routines	Parent-reported				x	x		x	x		x	x				
Night sleep duration	Parent-reported				x	x		x	x		x	x				
Bedtime	Parent-reported				x	x		x	x		x	x				
Night awakenings	Parent-reported				x	x		x	x		x	x				
Depression	Face-to-face interview	MFQ												x		
BPD symptoms	Face-to-face interview	UK Childhood Interview for DSM-IV BPD													x	
Psychotic symptoms	Face-to-face interview	PLIKSi														x

BPD=Borderline personality disorder; FAI=Family Adversity Index; CTS=Carey Temperament Scale; MFQ=Mood and Feelings Questionnaire; PLIKSi= Psychosis-Like Symptom interview

© 2020 Morales-Muñoz I et al. *JAMA Psychiatry*.

© 2020 Morales-Muñoz I et al. *JAMA Psychiatry*.

eTable 2. Differences in Sociodemographic and Clinical Variables Between Nonparticipating and Participating Subjects in the Study

	**Non-participating group in the study**		**Participating group in the study**		**Non-participating versus participating**	
	Mean	*SD*	*Mean*	*SD*	*OR (95% CI)*	*p*
Maternal age when born	27.73	4.13	29.05	4.16	1.01 (0.99, 1.02)	0.302
Birth weight, grams	3356.04	605.96	3415.35	543.49	1.00 (0.99, 1.01)	0.602
Family Adversity score	4.83	3.40	3.95	3.62	0.96 (0.94, 0.97)	<0.001
Emotional Temperament score	39.81	6.31	39.21	7.53	1.00 (0.99, 1.01)	0.513
Depression 10 years score	4.21	1.48	4.02	3.22	0.97 (0.95, 0.99)	0.006
	**Non-participating group in the study**		**Participating group in the study**			
	N	*%*	*N*	*%*		
Sex<br>Male / Female	4754 / 4194	53.13 / 46.87	3535 / 3667	49.08 / 50.92	0.77 (0.67, 0.88)	<0.001
Premature<br>Yes / No	531 / 7912	6.29 / 93.71	325 / 6877	4.51 / 95.49	0.91 (0.65, 1.28)	0.585
Childhood abuse<br>Yes / No	543 / 5063	9.69 / 90.31	674 / 5573	10.79 / 89.21	0.80 (0.64, 1.01)	0.063

© 2020 Morales-Muñoz I et al. *JAMA Psychiatry*.

eTable 3. Estimates of Total, Direct, and Indirect Effects

Total effects													
	**FAI**	**Child abuse**	**Premature**	**Sex**	**Maternal age born**	**Emotional temperament**	**Regular sleep routines 3.5y**	**Night waking18mo**	**Regular sleep routines 6mo**	**Bedtime 3.5y**	**Regular sleep routines 5.8y**	**Night sleep 3.5y**	**Depression 10y**
**Depression 10 y**	0.170	0.046	0.030	-0.042	-0.048	0.050	0.003	-0.031	-0.009	0.020	-0.041	-0.014	0.000
**BPD 11-12 y**	0.113	-0.003	0.026	0.000	-0.025	0.037	0.001	-0.009	-0.003	0.012	-0.012	-0.039	0.284
**Psychosis12-13 y**	0.091	0.018	0.007	0.053	-0.021	0.002	0.039	0.035	-0.055	0.003	-0.095	-0.002	0.153
**Direct effects**													
**Depression 10 y**	0.170	0.046	0.030	-0.042	-0.048	0.050	0.003	-0.031	-0.009	0.02	-0.041	-0.014	0.000
**BPD 11-12 y**	0.065	-0.016	0.017	0.012	-0.012	0.022	0.000	0.000	0.000	0.006	0.000	-0.035	0.284
**Psychosis12-13 y**	0.065	0.011	0.003	0.06	-0.013	-0.006	0.038	0.04	-0.054	0.000	-0.089	0.000	0.153
**Indirect effects**													
**Depression 10 y**	0.000	0.000	0.000	0.000	0.000	0.000	0.000	0.000	0.000	0.000	0.000	0.000	0.000
**BPD 11-12 y**	0.048	0.013	0.009	-0.012	-0.014	0.014	0.001	-0.009	-0.003	0.006	-0.012	-0.004	0.000
**Psychosis12-13 y**	0.026	0.007	0.005	-0.006	-0.007	0.008	0.001	-0.005	-0.001	0.003	-0.006	-0.002	0.000

FAI=family adversity index; Child abuse= childhood abuse; BPD=borderline personality disorder

© 2020 Morales-Muñoz I et al. *JAMA Psychiatry*.

eFigure. Causal Diagram to Represent Temporal Ordering, Including Confounders

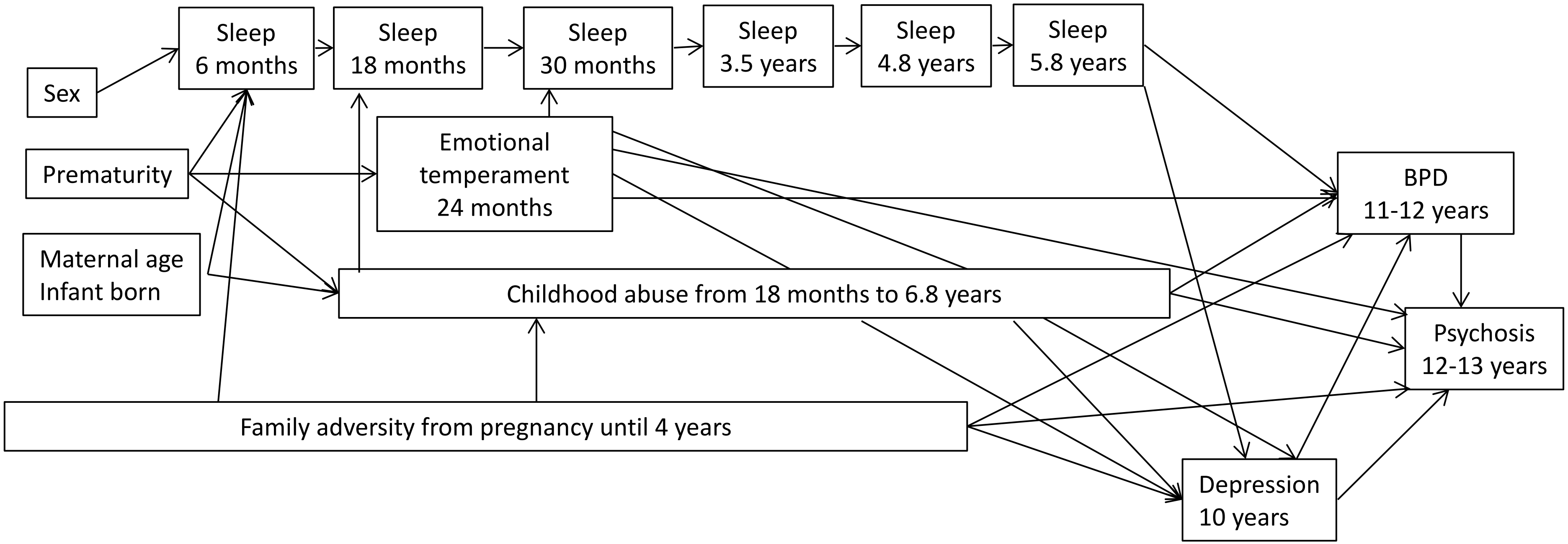

© 2020 Morales-Muñoz I et al. *JAMA Psychiatry*.
